# S57. TREATMENT SATISFACTION IN ACUTE PHASE PSYCHOSIS: COMPARISON BETWEEN ANTIPSYCHOTIC NAïVE AND PREVIOUSLY MEDICATED PATIENTS

**DOI:** 10.1093/schbul/sby018.844

**Published:** 2018-04-01

**Authors:** Lena Stabell, Rolf Gjestad, Rune Kroken, Else-Marie Løberg, Hugo A Jørgensen, Erik Johnsen

**Affiliations:** 1 Haukeland University Hospital; 2 Haukeland University Hospital, University of Bergen; 3 University of Bergen

## Abstract

**Background:**

Patient satisfaction is a complex phenomenon, and relations between patient satisfaction, symptom load, insight, side effects and depression have been shown in patients treated for psychosis. However, few studies on patient satisfaction in psychosis discriminate between persons previously treated with antipsychotic drugs and those who are antipsychotic drug naïve.

Our aim was to test whether predictors for treatment satisfaction differ between previously medicated with antipsychotics (PM) and those who were antipsychotic naïve (AN) at admission.

**Methods:**

In total 226 consecutive patients were included when admitted to hospital due to symptoms of active psychosis (≥4 on in one or more items of PANSS positive subscale), and were candidates for oral antipsychotic medication. At baseline PANSS, CDSS and Clinical Global Impression- Severity of Illness scale (CGI-S) were conducted. A total of 104 patients were assessed at discharge or follow up after a maximum of 11 weeks (mean 28.5 days, SD 14.1). In addition to the baseline assessments, patient satisfaction was assessed by the UKU Consumer Satisfaction Rating scale and the UKU Side Effect Rating Scale. For statistics structural equation modelling was performed to test multi sample growth models.

**Results:**

Patients assessed at baseline were found to be statistically similar to those also assessed at discharge/follow-up, with the exception of a slightly higher PANSS negative subscale score in those assessed at baseline only (independent samples t-test: p = 0.023, mean difference 2.3, 95% confidence interval of the mean difference 0.3–4.3).

There was a general improvement in function between baseline and follow-up, reflected in the CGI-S score reduction from 5.17 (SD .619) to 3.70 (SD 1.09) and symptom reduction in PANSS positive: mean change - 6.68 (SD 5.00).

For PM patients (N =55), satisfaction was predicted by level of insight (b = - 2.21, β = - 0.42, p = 0.000), and positive symptom reduction (b = -0.56, β = -0.39, p = 0.012). The most satisfied patients had high level of insight at baseline, and the steepest decline in positive symptoms.

For AN patients (N =49), satisfaction was predicted by level and change of insight (respectively: b= - 2.21, β = - 0.46, p = 0.000; b = - 1.53, β = - 0.32, p = 0.032), change in depression (b = -0.37, β = -0.26, p = 0.025), and side effects (b = -0.15, β = -0.30, p = 0.033). The most satisfied patients had high level of insight at baseline, most improvement in insight, the steepest decline in the CDSS score, and the lowest level of side effects.

**Discussion:**

The consecutive inclusions of patients make these finding relevant and generalizable to everyday practice in similar clinical settings.

Our findings suggest that reducing positive symptoms and side effects are important, but not solely sufficient to enhance patient satisfaction, and that differences exist among drug naïve and previously medicated patients. Improving insight and reducing depression are key processes to enhance satisfaction, particularly for antipsychotic naïve patients. Being drug naïve may be considered a proxy for First Episode Psychosis (FEP). Symptoms of depression seem to be particularly prevalent in FEP patients (1), and might dominate the experienced distress relative to symptoms of psychosis in these patients. Furthermore, FEP patients are more sensitive to the side effects of antipsychotic drugs. Another key target for improving satisfaction is to keep antipsychotic drug treatment at the lowest effective doses, as most side effects are dose-related.

**References:**

1. Cotton SM, Lambert M, Schimmelmann BG, Mackinnon A, Gleeson JFM, Berk M, et al. Depressive symptoms in first episode schizophrenia spectrum disorder. Schizophrenia research. 2012;134(1):20–6.

